# A Maturational Frequency Discrimination Deficit May Explain Developmental Language Disorder

**DOI:** 10.1037/rev0000436

**Published:** 2023-07-27

**Authors:** Samuel David Jones, Hannah Jamieson Stewart, Gert Westermann

**Affiliations:** 1 Department of Psychology, Bangor University; 2 Department of Psychology, Lancaster University

**Keywords:** developmental language disorder, auditory processing, spoken word recognition and retrieval, neural network, neural population geometry

## Abstract

Auditory perceptual deficits are widely observed among children with developmental language disorder (DLD). Yet, the nature of these deficits and the extent to which they explain speech and language problems remain controversial. In this study, we hypothesize that disruption to the maturation of the basilar membrane may impede the optimization of the auditory pathway from brainstem to cortex, curtailing high-resolution frequency sensitivity and the efficient spectral decomposition and encoding of natural speech. A series of computational simulations involving deep convolutional neural networks that were trained to encode, recognize, and retrieve naturalistic speech are presented to demonstrate the strength of this account. These neural networks were built on top of biologically truthful inner ear models developed to model human cochlea function, which—in the key innovation of the present study—were scheduled to mature at different rates over time. Delaying cochlea maturation qualitatively replicated the linguistic behavior and neurophysiology of individuals with language learning difficulties in a number of ways, resulting in (a) delayed language acquisition profiles, (b) lower spoken word recognition accuracy, (c) word finding and retrieval difficulties, (d) “fuzzy” and intersecting speech encodings and signatures of immature neural optimization, and (e) emergent working memory and attentional deficits. These simulations illustrate many negative cascading effects that a primary maturational frequency discrimination deficit may have on early language development and generate precise and testable hypotheses for future research into the nature and cost of auditory processing deficits in children with language learning difficulties.

There is astonishing variability in rates of early language development. Looking beyond population means, we see large windows of time in which language skills may emerge without any concern (Braginsky et al., 2018). Sometimes, however, a child’s language is delayed enough to cause alarm among personal and professional caregivers. An estimated 7.5% of English-speaking children find acquiring and using language difficult enough to potentially interfere with their day-to-day emotional well-being and later with their educational outcomes (Norbury et al., 2016). Where such difficulties are evident in the absence of any obvious biomedical cause, such as Down syndrome, the child may be diagnosed with developmental language disorder (DLD) and may undertake a tailored program of language intervention targeting their specific areas of difficulty (Bishop et al., 2016).

Language disorder identification, assessment, and intervention are challenging because of the significant heterogeneity seen among affected children. Any aspect of language may be disrupted in DLD, from phonology through to syntax and pragmatics, and children often show concurrent developmental difficulties, for instance in motor control, or comorbidity with conditions such as developmental dyslexia or attention-deficit/hyperactivity disorder (ADHD; Bishop et al., 2016). Furthermore, contrasting theoretical approaches have commonly centered on just one in a wide range of hypothesized cognitive faculties in accounting for discrete characteristics of this multifaceted profile. This approach has sometimes given the inaccurate impression that DLD is evidence of an isolated deficit in that faculty alone, for instance in working memory (Archibald & Gathercole, 2006), predictive processing (Hestvik et al., 2022), lateral inhibition (McMurray et al., 2019), or statistical learning (Ullman & Pierpont, 2005).

The complex symptomology seen in DLD and overlap across associated diagnostic groups, at the level of both linguistic profile (i.e., from phonemes to pragmatics) and implicated cognitive faculties (e.g., working memory, statistical learning), has fostered a shift toward a “transdiagnostic” mindset in neurodevelopmental disorder research (Astle et al., 2022). Here, focus is on what we might call canonical features of impairment—features sometimes termed “bridging symptoms”—that hold widely not just within but often across diagnostic groups. Working memory deficits measured, for instance, in the nonword repetition and span tasks are widely considered one such canonical feature of developmental disorder, given that such deficits appear quite consistently across young children with a range of developmental difficulties (Archibald & Harder Griebeling, 2016; Gray et al., 2019; Henry & Botting, 2017).

Maintaining that there are canonical features of developmental disorder is, of course, very different from assuming there is a single cause of any given disorder. In general, contemporary research on early language disorder is averse to the notion that the varied profiles seen among children might have a single cause. This is perhaps a well-justified reaction to early research that held up DLD as evidence of an isolated deficit in an innately specified language acquisition device (a “grammar module” of the brain encoded by the forkhead box P2 gene; Pinker, 1994) or similarly suggested that DLD was evidence of a discrete deficit in, for instance, working memory or statistical learning. We now know that the picture is considerably more complex. At the levels of genetics, neurobiology, and cognition, DLD appears to entail a constellation of causal mechanisms and risk factors (Bishop, 2006). A transdiagnostic, mechanism-centered approach fully appreciates this complexity and attempts to identify those dimensions of disorder that apply widely (though not uniformly) and which may point us to better understanding and more effective intervention strategies (Fletcher-Watson, 2022). The careful, in-depth study of a specific and well-recognized canonical area of difficulty might show us how much we “get for free” when we really explore the wide cascading effects implied by that area of difficulty.

The present study centers on one such canonical feature of DLD: auditory processing difficulties. While deficits in auditory perception are widely identified among children with neurodevelopmental disorder, most notably in DLD and dyslexia, the extent to which such deficits can explain early speech and language problems remains controversial (Bishop et al., 1999, 2012; Bishop & McArthur, 2005; Haake et al., 2013; McArthur & Bishop, 2004; Merzenich et al., 1996; Rosen, 2003; Tallal, 2013). In this study, we hypothesize that disruption to the maturation of the neural architecture underpinning high-resolution frequency discrimination from the prenatal period through the first 2 years of life (specifically, a disruption to basilar membrane maturation and resulting deficits in auditory brainstem optimization) may play a causal role in early speech and language disorder. Our account builds on prior work by McArthur and Bishop (2004) and Bishop and McArthur (2005) who first suggested that deficits in frequency discrimination may play an important role in the impairments observed among some children and adolescents with a diagnosis of DLD. In this study, we aim to substantially develop this account and to demonstrate its strength in a series of computational simulations that illustrate the varied consequences of a low-level frequency discrimination deficit within a controlled and transparent artificial learning environment. We aim to document the varied potential costs to early language development—that is, the many cascading effects that we “get for free”—as a result of a fundamental maturational deficit in frequency discrimination.

We begin this report by reviewing empirical research into the auditory processing skills of children with language disorder, highlighting an evolution from early theoretical accounts centered around temporal processing, which relates to the speed at which the auditory system responds to acoustic input, to relatively recent accounts centered around frequency or spectral processing. We then review research into the maturation of the neural architecture supporting high-resolution frequency discrimination ability from the prenatal and neonatal periods through childhood, before considering how a disruption to this typical maturational trajectory might give rise to speech and language deficits. Subsequently, we present a computational model in which we simulate different rates of maturation in frequency discrimination ability while monitoring language acquisition rates, spoken word recognition accuracy, proxies for word finding latency, and neural speech representation integrity. We then discuss the implications of our results, the limitations of our computational approach, and directions for future investigation.

## From Temporal to Spectral Processing Deficits in Language Disorder Research

A dominant view developed principally through the work of Tallal et al. is that children with language learning difficulties have a primary deficit affecting the perception of acoustic signals that change rapidly, something that these authors refer to as a temporal processing deficit^1^ (e.g., Merzenich et al., 1996; Tallal et al., 1981). Much of the empirical research in this direction made use of the auditory repetition task, or ART, in which children press buttons to identify changes in frequency in a series of pure tones. In the ART, performance accuracy among children with DLD was regularly shown to decrease significantly when interstimulus interval (ISI; i.e., the gap between tones) was reduced to below approximately 250 ms, lending apparent support to the hypothesis that these children’s auditory processing systems were ill-equipped to accurately perceive and encode rapidly unfolding natural speech (Merzenich et al., 1996; Tallal et al., 1981). This line of argument has been pursued in a significant body of research and has motivated the development of the Fast ForWord program of intervention, which claims to be able to train sensitivity to rapidly occurring auditory stimuli through the controlled manipulation of ISI and in doing so confer gains in speech and language abilities (Tallal, 2013).

Despite the initial dominance of the temporal processing deficit hypothesis, however, a series of failed replications, both of the basic research and of the Fast ForWord intervention (Strong et al., 2011; Bishop & McArthur, 2005; McArthur & Bishop, 2004; see Rosen, 2003, for review), has motivated the search for alternative characterizations of the auditory perceptual deficits that appear to affect many children with speech and language problems. One promising, though comparatively underexamined, view is that such deficits are spectral rather than temporal in nature (Bishop & McArthur, 2005; McArthur & Bishop, 2004; Mengler et al., 2005). That is, for many children the difficulty relates principally to distinguishing discrete sounds of similar frequency rather than discrete sounds that rapidly follow one another. For instance, across two studies, Bishop and McArthur presented children aged 10–19 with and without language disorder with a baseline tone of 600 Hz and a distinct tone, which was initialized at 700 Hz, but which was raised or lowered by increments of 25 Hz to determine the minimal frequency discrimination threshold, or limen, that participants could identify (Bishop & McArthur, 2005; McArthur & Bishop, 2004; see also Mengler et al., 2005). These authors found that the minimal frequency discrimination threshold among children with severe language disorder was 750 Hz (i.e., a 150-Hz disparity) during an initial assessment and 674 Hz at follow-up (i.e., a 74-Hz disparity) compared to 629 and 624 Hz disparities, respectively, for control children. Readers may wish to visit one of the many freely available online pure tone generators to compare tones in this range themselves. For many, the average difference between the minimal threshold tones identified by children with DLD (i.e., 600 and 750 or 674 Hz) will appear striking, attesting to the difficulty such a deficit may cause during the analysis of the complex spectral profiles of natural speech (Nuttall et al., 2018; Sumner et al., 2018).

Crucially, Bishop and McArthur found that this deficit in frequency discrimination was observed regardless of the rate of stimulus presentation, providing compelling evidence that the auditory processing difficulties of some children affected by language disorder are spectral rather than temporal in nature and perhaps explaining the failed replications of key studies in the temporal processing deficit literature (Bishop & McArthur, 2005; McArthur & Bishop, 2004; Mengler et al., 2005; Rosen, 2003; Strong et al., 2011). What is more, even those children with DLD who performed well in the behavioral tone discrimination task nevertheless showed immature waveforms during electroencephalography (EEG) monitoring, providing tentative support for the maturational account that Bishop and McArthur (2005) then offer to explain their findings.

## The Maturation of Frequency Discrimination Skills

Bishop and McArthur (2005) explained their results in terms of a disruption to the typical maturation of high-resolution frequency discrimination. In order to situate this account, upon which we intend to elaborate, it is useful to review key research on the early maturation of frequency discrimination skills and the neural basis of these skills. In younger children and infants, probing the maturation of frequency discrimination skills presents a significant challenge. Paradigms such as head turning and high-amplitude sucking have provided mixed results and are open to interpretation, not least that a failure to discriminate tones in such paradigms may be the result of immature motor skills or attention (see Burnham & Mattock, 2014, for review). In response, some researchers have advocated the use of neuroimaging methods such as EEG and magnetoencephalography when studying frequency discrimination in neonates and infants (e.g., Novitski et al., 2007). Despite their own limitations, such neuroimaging methods are often considered to provide an index of neural activity that is relatively independent of motor and attentional factors (Novitski et al., 2007).

Neuroimaging involving neonates and infants corroborates indications from behavioral research of an early maturation in frequency discrimination ability (Jensen & Neff, 1993; Lopez-Poveda, 2014; Novitski et al., 2007; Shafer et al., 2000; Tharpe & Ashmead, 2001). This maturation is not uniform. High-frequency tone discrimination is approximately adult-like in apparently typically developing infants by 6 months of age. In contrast, low-frequency discrimination, in the range more regularly associated with speech signals (e.g., 400 Hz), develops more slowly, with continued maturation apparent in children up to ages 7–9 (Burnham & Mattock, 2014; Jensen & Neff, 1993). While the empirical data vary somewhat, estimates from the “odd-one-out” paradigm (also known as the “mismatch negativity paradigm”) suggest that newborns can detect a 20% though not a 5% change in frequency in a 250–4,000 Hz window (Novitski et al., 2007; see Burnham & Mattock, 2014, for review). Such findings support the view that frequency resolution improves considerably from birth through childhood, making it increasingly easy to discriminate competing acoustic signals and thus to perform the complex spectral analysis that accurate and efficient natural speech perception and encoding requires (Nuttall et al., 2018; Sumner et al., 2018).

**Figure 1 fig1:**
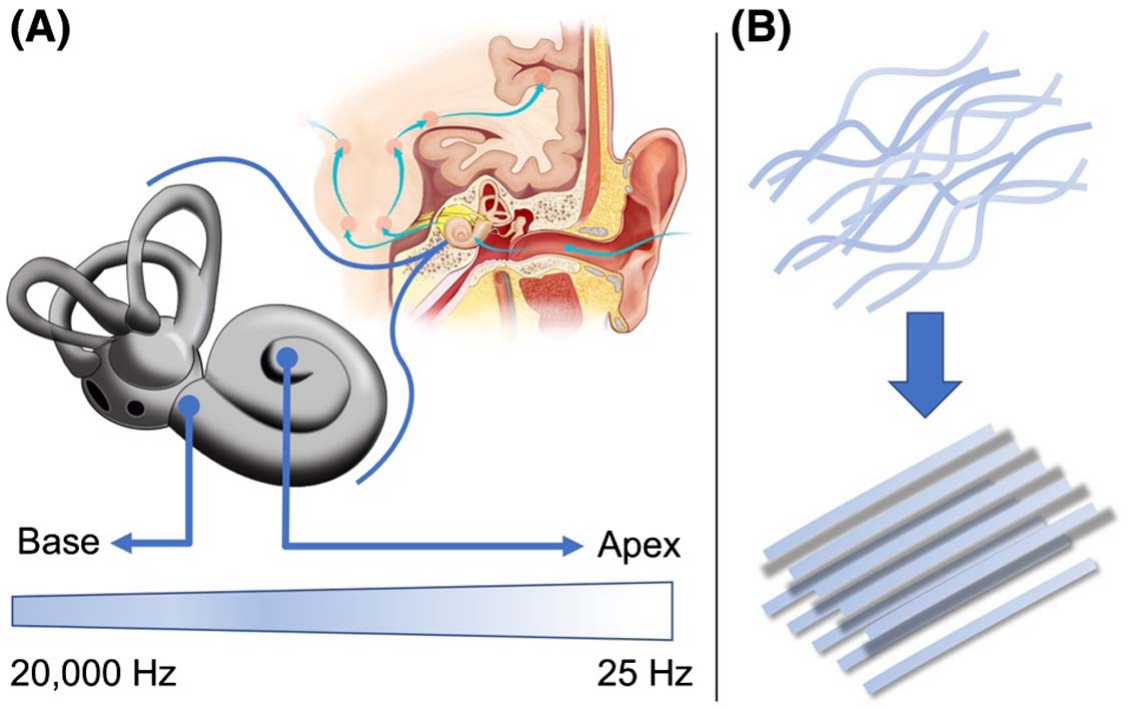
Schematic of Frequency Tuning and Structural Development in the Mammalian Cochlea *Note*. Panel A shows the location of the cochlea in the inner ear (colored inset), its coiled structure (in gray), and the mechanical frequency sensitivity gradient from base to apex of the basilar membrane within the cochlea. Panel B illustrates the development of basilar membrane microstructure supporting high-resolution frequency tuning, from fibers that are low diameter, sparse, and “braided” to fibers that are higher diameter, dense, and regular. The Panel A auditory system image (colored inset) is in the public domain (https://commons.wikimedia.org/wiki/File:Hearing_mechanics.png). The Panel A grayscale cochlea image is available under a Creative Commons Attribution Share Alike 4.0 International license (https://commons.wikimedia.org/wiki/File:Inner_ear.png). See the online article for the color version of this figure.

The maturation of frequency discrimination skills reflects changes in neural architecture that, though many important questions remain, are now in a large part reasonably well understood. A key characteristic of the auditory perceptual system upon which speech representation and use is based is its tonotopic structure. That is, throughout the auditory pathway, from the inner ear to the auditory brainstem and on to the auditory cortex, we see selective responsivity to acoustic input of particular frequencies among sensory cells and neurons that constitute the neural basis of frequency resolution and the decomposition of auditory signals, including speech (Echteler et al., 1989; Nuttall et al., 2018; Sumner et al., 2018). The characteristic “tonotopic” structure of the auditory pathway results predominantly from the physical properties of the basilar membrane, a 35-mm coiled membrane within the inner ear (Figure 1A).[Fig fig1]

The basilar membrane is narrow and firm at its base and as a result of these physical properties, fibers in this basal region vibrate maximally to the high frequencies in auditory input (Figure 1A; Sumner et al., 2018). The apex of the basilar membrane is, in contrast, wide and relatively slack and as a result, fibers in this apical region vibrate maximally to the low frequencies in auditory input (Figure 1A; Sumner et al., 2018). For instance, voiceless fricatives such as/ʃ/, which contain relatively high-frequency components, may stimulate basal regions of the membrane, while vowels such as /ɑ:/, which contain low-frequency components, may stimulate apical regions. Upon the basilar membrane sit a single row of approximately 3,500 inner hair cells, which become selectively responsive to specific frequencies—that is, they are “frequency-tuned”—as a result of their position on the basilar membrane (Sumner et al., 2018; Tani et al., 2021). In turn, inner hair cells are innervated by spiral ganglion neurons, which project to the cochlear nucleus, with this and subsequent innervation conserving tonotopic sensitivity and resulting in the emergence of frequency sensitive “maps” throughout a complex array of subcortical structures of the auditory brainstem and on to the peripheral auditory cortex. The physical properties of the basilar membrane are, therefore, at the heart of frequency sensitivity and acoustic signal decomposition across the auditory pathway, and this itself underpins accurate and efficient speech processing and encoding (Burnham & Mattock, 2014; Echteler et al., 1989; Nuttall et al., 2018; Sumner et al., 2018; Tani et al., 2021). From the third trimester to 6 months of age, structures from the auditory nerve throughout the auditory pathway to the auditory cortex undergo substantial changes in synaptic organization, myelination, and dendritic arborization, and this process of maturation continues through 2 years of age during a typically rich period of language development (Chonchaiya et al., 2013). Work by Chonchaiya et al. (2013) indicates that, by 9 months of age, auditory brainstem responses continuous with relatively mature brainstem organization are predictive of better language outcomes.

Recent research has cast light on how the pre- and postnatal structural development of the basilar membrane underpins the emergence of high-resolution frequency tuning across the auditory-linguistic pathway. Studies using electron microscopy and polarized light microscopy have shown that the basilar membrane is composed of collagenous filaments, or fibers, which are initially relatively low diameter, sparsely organized, and “braided,” but which increase in diameter, density, and linear regularity throughout early development (Figure 1B). Such studies have also determined an uneven time course in which structural maturation is slower in the membrane apex than it is in basal regions, a finding consistent with behavioral and neurophysiological evidence that low-frequency component tuning comes online relatively slowly (Burnham & Mattock, 2014; Novitski et al., 2007; Tani et al., 2021). Animal models also provide mounting evidence that the protein coding gene *emilin2* (elastin microfiber interfacer 2), which is part of the emilin family of glycoproteins that contribute in part to tissue elasticity, can seriously disrupt fiber development in the basilar membrane—that is, can curtail typical increases in fiber diameter, density, and linear regularity—and can, therefore, disrupt the membrane’s capacity to propagate frequency sensitivity throughout posterior structures of the auditory pathway supporting accurate and efficient frequency decomposition (Amma et al., 2003; Russell et al., 2020; Tani et al., 2021). This literature demonstrates how a genetic abnormality can in principle disrupt the emergence of the mechanical gradient of the basilar membrane.

## Toward a Maturational Account of Frequency Resolution Deficits and Speech and Language Difficulties

Before stating our hypothesis, let us take stock of the key points reviewed so far:

1 Auditory processing deficits are widespread among children with DLD, and these deficits may be frequency-based rather than temporal in nature.
2 Evidence that deficits are related to frequency analysis points to specific cellular and neural structures of the auditory pathway. Specifically, the basilar membrane is at the heart of frequency tuning across the auditory pathway, with tonotopic maps emerging throughout the auditory brainstem and cortex predominantly as a result of dynamic adaptation to the structural properties—that is, the mechanical gradient—of the basilar membrane.
3 The basilar membrane undergoes crucial structural changes during prenatal development, with the fibers from which the membrane is composed increasing in diameter, density, and regularity. This process of maturation is integral to the emergence of tonotopic sensitivity across the auditory pathway.

Our hypothesis is, then, that:

Early disruption to the maturation of the physical properties of the basilar membrane, which underpin that membrane’s mechanical gradient (i.e., increases in fiber density, diameter, and linear regularity), may disturb the optimization of the posterior auditory pathway from the brainstem to the cortex, curtailing high-resolution tonotopic sensitivity and contributing to speech and language difficulties in some children.

The auditory pathway is, of course, a highly complex system, which could be disrupted by any number of influences operating across any number of its subsystems or processes. It is, for instance, possible that auditory brainstem and auditory cortex optimization are disrupted despite a properly maturing basilar membrane and a properly functioning inner ear more generally. A range of such alternative possibilities are presented in our Discussion section. Nevertheless, we believe that the hypothesis above provides a strong starting point for systematic investigation given that (a) the auditory processing deficits we see in DLD appear to be spectral in nature and (b) that a properly matured basilar membrane sits at the heart of high-resolution frequency processing across the auditory pathway. Our hope is that this literature review has shown that—though more work is undoubtedly required—there already exists a great deal of empirical evidence bearing on typical and atypical auditory pathway maturation and the potential impact of a low-level maturational problem in this pathway on the emergence of speech and language. In our view, what is currently required to direct future investigation is a compelling theoretical account linking these fragmentary research strands and this is what we attempt to provide in the present study. Our aim is emphatically not to suggest that frequency discrimination deficits wholly explain early language disorder. Instead, we aim to flesh out one candidate mechanistic pathway within a complex constellation of many.

In what follows, we simulate and monitor the dynamic adaptation of an artificial auditory-linguistic pathway (broadly auditory brainstem to cortex) in response to biologically plausible representations of speech-elicited activation patterns in the developing cochlea, under (a) nondevelopmental, (b) regular, and (c) delayed maturational trajectories. We show how a disruption to the maturation of cochlea microarchitecture may result in the atypical optimization of subsequent neural pathways, qualitatively accounting for several commonly recorded characteristics of atypical human linguistic behavior and neurophysiology, namely, (a) delayed language acquisition profiles (e.g., Norbury et al., 2016), (b) spoken word recognition deficits (Andreu et al., 2012; Evans et al., 2018; Rispens et al., 2015; Velez & Schwartz, 2010), (c) word finding or retrieval problems (Kambanaros et al., 2015; Messer & Dockrell, 2006), (d) “fuzzy” long-term speech representations (Claessen et al., 2009), (e) atypical neural signatures of auditory signal processing (e.g., Bishop & McArthur, 2005), and (f) apparent working memory deficits, attributable, we argue, to the imprecision of activated long-term speech representations (Henry & Botting, 2017; Jones & Westermann, 2022).

## Overview of Simulations

### Network and Training and Testing Regimes

The architecture used in these simulations is an artificial neural network known as a deep convolutional neural network. The work of McDermott et al. has been instrumental in demonstrating that despite obvious disparities between the biological auditory pathway and this artificial counterpart, including in general complexity and in learning procedures (see Discussion), close parallels are observed between convolutional neural network activity and human behavioral and neural responses across a wide range of tasks, such as speech localization, pitch perception, and hearing in noise (Francl & McDermott, 2022; Kell et al., 2018; Saddler et al., 2021). Convolutional neural networks are not “circuit models” of the brain. That is, these networks are not intended to explicitly model fine-grained physiology such as ion channel behavior (e.g., see Higgins et al., 2017, for a circuit model of speech perception and category formation). Rather, convolutional neural networks can provide high-order “computational” insight, in the sense of Marr (1982), into how a perceptual processing hierarchy dynamically adapts to a particular form of input to solve a certain problem under varying constraints.^2^

**Figure 2 fig2:**
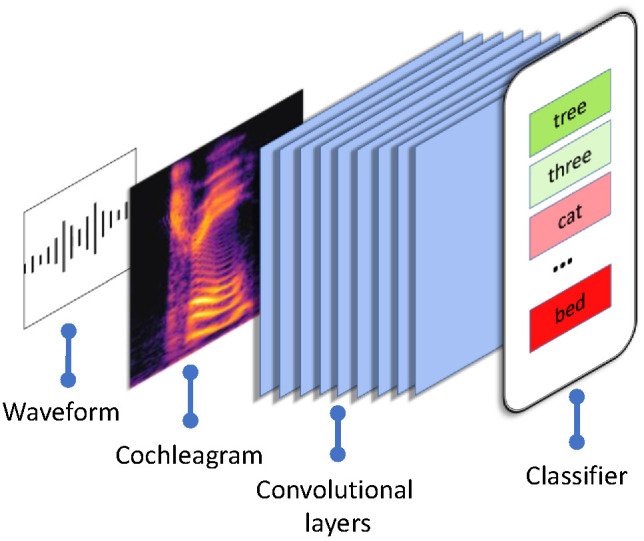
Neural Network Schematic *Note*. Authentic, raw spoken waveforms are first passed through a cochlea model, before being passed through the deep convolutional neural network and the 35-way classifier. See the online article for the color version of this figure.

Our simulations made use of the ResNet-18 deep convolutional neural network (He et al., 2015), which we implemented using PyTorch (Paszke et al., 2019) in Python (Python Software Foundation, 2008). A full network description can be retrieved by running the Jupyter Notebook associated with this project. Note that following the code examples associated with Stephenson et al. (2020; see https://github.com/schung039/neural_manifolds_replicaMFT), many of our analyses center around the networks’ 20 convolutional layers. For this reason, these layers are detailed in the Appendix alongside key hyperparameters. A total of nine convolutional neural networks (*n* = 3; conditions defined below) were trained and tested on spoken words from the speech commands data set (Warden, 2018), which contains 105,829 one-second spoken word waveforms of 35 word types (Figure 2). The speech commands data set was chosen for this project because it is free and openly available and because it is perhaps unique in comprising such a large number of exemplars of natural speech. Limitations of the speech commands data set are noted in our discussion.[Fig fig2]

Over 10 cycles, or “epochs,” of training, networks were required to categorize each spoken word that they perceived by outputting a probability distribution over their 35-word lexicon. The word with the highest probability assigned was taken as the networks’ selection. Networks responded dynamically to error signals propagated upon an incorrect classification by updating their inner weight matrices using the backpropagation algorithm after each spoken word exposure (i.e., batch size = 1) in order to reduce the future error rate. This constitutes a broad computational analogy to fluctuation in synaptic connection strength due to long-term potentiation (Lillicrap et al., 2020). Throughout training, networks were presented with random samples of 4,000 exemplars per epoch from the speech commands data set. Random samples were matched within epochs across the network groups we define below. For instance, Network 1 in each experimental condition saw the same random samples of training data, which differed in each training epoch. This ensures that any later observed group-level performance discrepancies are not a function of differences in the data that the network has been trained on. We want to emphasise that there is nothing special about the word as a unit of representation here. Our choice of data set principally reflects its scale and the fact that it contains authentic speech, and similar effects would be expected were we modeling phonemes or multiword constructions.

Later, at test, neural networks were presented with another random sample of 1,000 words from the speech commands data set, a random sample which was again matched across conditions (defined below). We recorded a range of test performance metrics, including speech recognition accuracy, proxies for response latency and word finding difficulties (namely, predictive distribution entropy or spread), confusion matrixes, and item-specific effects (i.e., fitting a Bayesian model of what lexical features contributed to a correct or incorrect spoken word classification). We also analyzed what form the networks’ internal speech representations took, using a statistical physics method known as mean field theory-based manifold analysis to measure the average degree of spread of a single neural representation and its overlap with competitor representations. These techniques are described in more detail below.

**Figure 3 fig3:**
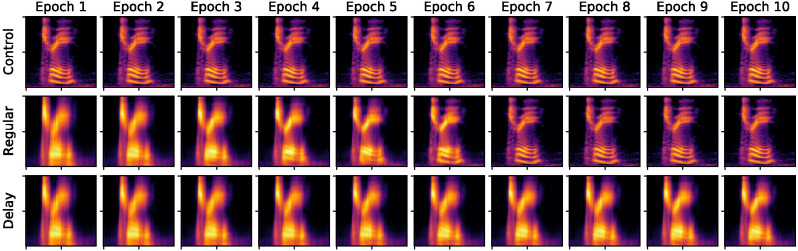
Schedules of Simulated Basilar Membrane Maturation *Note*. Shown is a cochleagram of the word tree under varying rates of maturation in spectral (i.e., *y-*axis) acuity within three conditions (control, regular, delay) and across 10 cycles (epochs) of training. See the online article for the color version of this figure.

Convolutional neural networks are, in the vast majority of research, configured “a-developmentally.” That is, parameters such as the number of layers or number of neurons per layer, etc., are fixed at the outset and remain static during network training and testing (cf. Westermann et al., 2006; Westermann & Jones, 2021;Westermann & Ruh, 2012; these studies similarly involve neural networks that change structurally during learning, e.g., in terms of the number of hidden units that they have). In contrast, one innovation of the present study was to model the maturation of high-resolution frequency discrimination skills using what is known as scheduled learning. That is, we ran distinct populations of neural networks in which frequency discrimination ability matured at different rates, according to different schedules across 10 epochs of training. As can be seen in Figure 2, raw spoken word waveforms were initially passed through a cochleagram model developed specifically to replicate typical, human cochlea function (McDermott & Simoncelli, 2011). The resultant 100 × 100 dimension cochleagram images were then passed through the deep convolutional neural network and later into a 35-way classifier. In three discrete conditions, we manipulated the maturation of that initial cochleagram model in three neural networks (*n* = 3, *N* = 9). Networks 1, 2, and 3 in each condition had identical weight initializations. This ensured that any group-level performance discrepancies observed were not a function of the networks’ starting states. Condition 1 was a-developmental—that is, a baseline or control network—meaning that this network received high-resolution speech input from the outset and no changes to the network occurred during 10 epochs of training (see Figure 3, Row 1). In contrast, the cochlea models of networks in Conditions 2 and 3 maturated according to a specific schedule. In Condition 2, frequency resolution started low, but improved rapidly, resulting in full-resolution processing (i.e., baseline equivalent acuity) by Epoch 7 (Figure 3, Row 2). This can be seen in the increasing *y*-axis acuity (i.e., decreasing vertical blur) across the cochleagrams in Row 2 of Figure 3. Networks in Condition 3, in contrast, started with precisely the same standard of frequency resolution as the networks in Condition 2—that is, frequency resolution is identical during training Epoch 1 in the regular and delay conditions—but then followed a delayed maturation schedule, never reaching baseline acuity (Figure 3, Row 1). In both the delay and regular conditions, frequency resolution was constrained using a normalized box filter with a kernel of shape (1, *y*), where *y* decreased at different rates over 10 epochs: from 25 to 1 in the regular condition and from 25 to 16 in the delay condition.[Fig fig3]

### Methods of Analysis

All postsimulation analyses were conducted in R (RStudio Team, 2016). During training and testing, networks were presented with cochleagrams and in response output probability distributions over their 35-word lexicons. The word assigned the highest probability was taken as a network’s classification and where this corresponded to the true target cochleagram a “hit” was scored. The analysis of our training data involved measuring spoken word classification accuracy by training epoch. At test, we measured classification accuracy and the average maximum probability and probability distribution entropy output when a classification was made. These metrics provide a proxy for a network’s certainty in its classifications. A high probability, low entropy (i.e., low spread) distribution signals high certainty in a judgment, while a low probability, high entropy (i.e., high spread) distribution signals low certainty in a judgment.

We then teased apart item-specific effects, looking for subsets of words on which regular or delayed networks performed better or worse. As part of this analysis into item-specific effects, we ran a Bayesian regression model (Burkner, 2017) in which the percentage of correct classifications per word was predicted by condition (i.e., regular, delayed) in interaction with two relevant independent variables that have generated considerable interest in developmental psycholinguistics: word frequency and word phonological neighborhood density (e.g., Ambridge et al., 2015; Jones & Brandt, 2019; Rispens et al., 2015). Word frequency quantifies how common the word is in the exposure language; here, the speech commands corpus from which training words were randomly sampled. Phonological neighborhood density meanwhile quantifies the average distance, calculated on the basis of phonological transcriptions, between each word and the other 34 words in the training data. Relatively high input frequency is regularly associated with better language learning in children (Ambridge et al., 2015), while high phonological distance (i.e., phonemic dissimilarity) may improve speech classification accuracy among human listeners because potential between-item confusion is lower (Karimi & Diaz, 2020). As our modeling approach did not involve semantic representations, it was not possible to include other variables of potential interest such as word concreteness, valence, or relevance to infants and babies (Braginsky et al., 2018; Jones & Brandt, 2019).

Artificial neural networks are sometimes criticized for being inscrutable “black boxes.” Yet, there exist numerous methods that enable the researcher to go beyond performance metrics such as accuracy alone to peer inside the network and understand how it is representing information in the service of completing a certain task. Exploiting such methods is vital to the present study because our interest is in how a processing hierarchy modeling the auditory pathway from brainstem to cortex optimizes in the face of low-level constraints on frequency tuning in the cochlea. Convolutional neural network activation patterns have been shown to align broadly (i.e., not on a layer-to-structure level of granularity) with activation patterns in the biological brain (Kell et al., 2018; cf. Thompson, 2020). Furthermore, Bishop and MacArthur’s work in this direction shows that even when there is apparently no group difference in performance metrics such as accuracy, frequency resolution deficits may be associated with different neural signatures across groups with and without language disorder (Bishop & McArthur, 2005; McArthur & Bishop, 2004). Similarly, Chonchaiya et al. (2013) showed that auditory brainstem responses continuous with immature brainstem optimization predict relatively poor language outcomes. We wondered whether a similar neural signature of auditory processing impairments within the context of language learning deficits would emerge within our computational framework.

**Figure 4 fig4:**
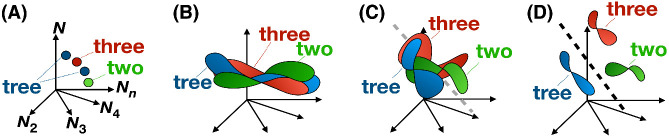
Principles of Neural Population Geometry *Note*. Panel A shows the spoken words tree, three, and two as response vectors in high-dimensional space, with axes *N*_1_ to *N*_*n*_ representing the response of a specific neuron within the population in spikes per second. The population here could be any structure within the auditory pathway (e.g., inferior colliculus, medial geniculate nucleus). Note that response vectors can also be shown as trajectories over time (e.g., see Chung & Abbott, 2021). Exemplars of the same word, for example, tree, reside in a different neural response vector as a function of neural noise and speaker and context effects, but collectively form a quasi-continuous manifold. (Note that in a deviation from the mathematical definition of a manifold, neural manifolds need not be smooth and continuous, but are instead held to comprise the convex hull of the distribution of neural responses elicited by a fixed class of stimulus). Panels B–D illustrate the neural basis of the well-studied transformation across the auditory system from noise-sensitive to speech-selective responses (e.g., Davis & Johnsrude, 2003; DeWitt & Rauschecker, 2012; Kaas et al., 1999; Okada et al., 2010). Early in the auditory pathway manifolds of different speech strings intersect substantially due to cellular responsiveness to low-level auditory features. Intersecting manifolds are then incrementally untangled and reduced in dimensionality across the auditory pathway. Panel C shows an intermediate, “low-capacity” system in which residual manifold tangling is evident. Panel D shows an optimal system with distributed speech representations that accommodate variability in the speech stream, but which are discrete and amenable to form the focus of attention. The dotted line in panels C and D illustrates a simulated attentional mechanism (implicated in both recognition and retrieval), which is overwhelmed (Panel C) or effective (Panel D) as a function of the precision of activated long-term memories. Adapted from “Under-Resourced or Overloaded? Rethinking Working Memory and Sentence Comprehension Deficits in Developmental Language Disorder,” by S. D. Jones and G. Westermann, 2022, *Psychological Review*, *129*(6), pp. 1358–1372 (https://doi.org/10.1037/rev0000338). Copyright 2022 by The Author(s). CC BY. See the online article for the color version of this figure.

To better understand how our neural networks dynamically optimized to cochlea representations with varying spectral acuity (Figure 3), we used a recently developed framework known as mean field theory-based manifold analysis (MFTMA; Figure 4; Chung & Abbott, 2021; Chung et al., 2018; Cohen et al., 2020). Under this approach, each neuron in any given structure of the auditory pathway, for instance the inferior colliculus, is configured as a single axis against which the spiking activity in that neuron can be plotted. Collectively, neurons in a given neural structure then define a neural state space (Figure 4A; graphically, a collection of axes) in which patterns of activation can be plotted either as trajectories through time or averaged spikes-per-second vectors. Given neural noise and variability in speaker and communicative context, no two instances of any given speech string stimulate the same response vector within that neural state space, that is, repeated spoken instances of a given linguistic structure never stimulate each neuron in the state space to the same degree. Repeated exposure to a range of exemplars from a single linguistic class, whether phoneme, word, or construction, therefore, stimulates a unified population response known as a “manifold,” which is a quasi-continuous subspace of the neural state space that can be considered the neural basis of the representation of that class (Cohen et al., 2020). Implicitly estimating the bounds of this neural manifold is considered integral to recognizing and producing novel yet valid speech, as if recognizing that instances of this class may regularly stimulate activation patterns within but not substantially outside this region of the state space (Cohen et al., 2020; DiCarlo & Cox, 2007; Stephenson et al., 2020; Yamins & DiCarlo, 2016).[Fig fig4]

The major contribution of the MFTMA method is enable us to treat distributed biological and artificial neural activation patterns as continuous geometric shapes that we can measure. Essentially, the convex hull of the collected response vectors (i.e., the points in Figure 4A) elicited by a fixed class of stimuli is treated as a single geometric object. In the present study, we are interested in two geometric quantities of neural representation that have received significant attention in the computational neuroscience literature. First, we are interested in the dimensionality of the pattern of activation (i.e., the manifold) underpinning responses to a certain class of spoken words (i.e., all instances of “tree”). That is, we are interested in how spread out through the neural state space speech representations are. Second, and relatedly, we are interested in the overlap between competitor neural representations, such as those underpinning the phonologically similar words “tree” and “three”. Within the MFTMA literature, overlap is quantified in terms of classification capacity, which is derived by calculating the number of speech manifolds that can be linearly separated from all competitor representations and standardizing the result by network layer size. In a low-capacity system, representations are highly overlapping (i.e., discrete representations involve activity in shared neurons), and the system struggles to use a linear separator to recognize or retrieve any single representation given this overlap (Figure 4B and C). In a high-capacity system, representation dimensionality (and other highly correlated quantities, including manifold radius) has been reduced to a level at which overlap is low and linear separation is more straightforward (Figure 4D).

With these properties in mind, Jones and Westermann (2022) drew a parallel between variance in a network’s classification capacity and the demands placed on human working memory or attentional systems as a function of the precision of activated long-term memories. Activated low-precision long-term memories, that is, memories with high dimensionality, place high demands on the system and compromise efficient processing, overwhelming working memory and attention (Figure 4C). On the other hand, activated high-precision long-term memories, that is, memories with low dimensionality, place low demands on the processing system because procedures, including speech recognition and retrieval, are facilitated if the target representation is relatively discrete (Figure 4D).

Research in this area, both computational work and work involving humans, points to potentially domain general transformations in representational structure from low-level structures such as the auditory nerve to high-level structures such as the peripheral auditory cortex. Broadly, low-level structures are noise sensitive, and so manifolds show extensive overlap (i.e., high dimensionality representations in a low-capacity system). However, within both biological and artificial neural processing hierarchies, architectural features such as pooling functions (where, for instance, a single neuron fires if any connected antecedent neuron fires) mean that early noise-sensitive representations become increasingly speech selective (Davis & Johnsrude, 2003; DeWitt & Rauschecker, 2012; Kaas et al., 1999; Okada et al., 2010; Yamins & DiCarlo, 2016). That is, we go from high-dimension representations in a low-capacity system early in the pathway to low-dimension representations in a high-capacity system late in the pathway. The neural population geometry view of this trajectory is illustrated in Figure 4, Panels B–D. Jones and Westermann did not present a maturational account of frequency resolution in the peripheral auditory system and speech deficits. Instead, their interest was on explaining variance in working memory task performance. However, these authors show that the trajectory shown in Figure 4 could be disrupted by the addition of broad Gaussian noise to input representations. Here, we intend to build substantially on this work by (a) using cochleagrams developed expressly to simulate human auditory physiology and (b) manipulating cochleagrams during training in line with known trajectories in the maturation of frequency discrimination skills, something we believe to be unique to the present study.

It is worth noting that we are using a powerful neural network with a large number of training samples of a relatively small number of word types. In general, these are perfect conditions for training a highly robust neural network that copes well in the face of input noise. Our intention throughout this project was to keep our manipulation subtle in line with the notion of a possibly subtle derailment of a typical maturational trajectory. Indeed, looking at Figure 3, it is clear that the cochleagrams in Epoch 10 retain something of a recognizable contour across conditions, and it might not be too challenging to visually identify this particular word, tree, from the cochleagrams of certain other words within the 35-word cohort. We did not, therefore, expect dramatic differential effects in the region of, for instance, 25% performance accuracy, which is the sort of disparity sometimes seen in empirical studies using so-called “extreme-group designs,” which compare quite severely language-impaired children to children with strong language skills (see West et al., 2017, for a criticism of this approach). Instead, we were looking for potentially subtle but consistent disparities in network optimization indices and behavior across conditions that align well with current behavioral and neurophysiological evidence from children with and without language learning difficulties.

## Results

### Classification Accuracy, Probability, and Entropy

**Figure 5 fig5:**
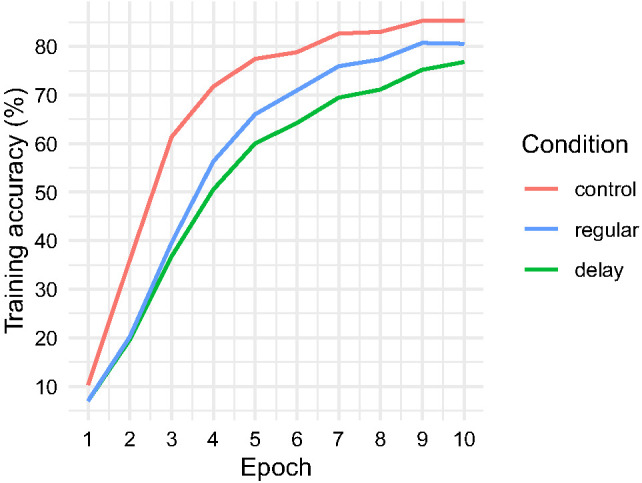
Training Accuracy by Epoch and Condition *Note*. See the online article for the color version of this figure.

In the analyses that follow, network performance is collapsed and reported as a condition mean. Spoken word classification accuracy by condition and training epoch is shown in Figure 5. Across epochs, networks in the optimal, a-developmental control condition outperformed the developmental networks in both regular and delay conditions. Constraining the maturation of high-resolution frequency, discrimination according to the schedules shown in Figure 3 promoted a clear disparity between regular and delay networks, with the regular networks performing better after Epoch 2 and this gap widening in line with the disparity in the resolution of spectral information generated by the networks’ cochlea model (Figure 3).[Fig fig5]

By Epoch 10, accuracy averaged 85.3% in the control condition, 80.6% in the regular condition, and 76.8% in the delay condition. A similar pattern was observed at test, where speech classification accuracy averaged 85.1% in the control condition, 83.9% in the regular condition, and 79.6% in the delay condition. During training and at test, accuracy reflects the networks’ ability to correctly classify spoken word cochleagrams. The difference between these analyses is that training-phase accuracy describes a learning trajectory, while test-phase accuracy reflects a cross-sectional analysis that is conducted when training is complete.

The accuracy data above represents a record of hits as a proportion of total exposures. However, it is also possible to get a picture of the networks’ confidence in their predictions by analyzing the maximum probability assigned to a prediction and the entropy (or spread, in bits) of the probability distribution output. This analysis indicated greater uncertainty in the predictions made by networks in the developmental conditions than in the optimal condition and greatest uncertainty in networks in the delay condition. Mean maximum probability assignment stood at 86.7% in the control condition, 81.5% in the regular condition, and 78.6% in the delay condition, while entropy or distribution spread in bits stood at 0.443 control, 0.612 regular, and 0.693 delay (i.e., indicating increasingly spread out predictive distributions). A similar pattern was observed when limiting our analysis to hits only: mean maximum probability assignment = 91.4% control, 87.2% regular, and 85.3% delay; entropy in bits = 0.306 control, 0.449 regular, and 0.496 delay.

In summary, networks in the maturational delay condition not only performed significantly less accurately than comparison networks, but also output relatively broad, highly spread probability distributions over their lexicons, considering many competitor words and assigning the true target relatively low probability even when accurate. Therefore, neural networks with maturational deficits in frequency resolution take longer to encode speech information, and metrics of test performance (i.e., low maximum probability, high entropy) suggest that formed speech encodings are inefficiently organized. In response to speech input, more of what we might consider the networks’ long-term memory (i.e., the fixed 35-word lexicon) becomes activated (i.e., we see high-spread predictive distributions), and the true target may be swamped in activated competitor representations. Qualitative analogies might be seen here between network performance and the DLD literature showing (a) delayed acquisition profiles (Norbury et al., 2016; a parallel with the disparity in network accuracy over training epochs), (b) lower spoken word recognition accuracy (Andreu et al., 2012; Evans et al., 2018; Rispens et al., 2015; Velez & Schwartz, 2010; a parallel with the network test phase accuracy disparity), and (c) word finding difficulties and residual uncertainty even when performing accurately, as evidenced, for instance, in eye tracking paradigms (Kambanaros et al., 2015; McMurray et al., 2019; Messer & Dockrell, 2006; a parallel with high entropy, low probability activation patterns). Later, we examine the representational basis of these performance profiles. First, however, we aimed to determine the particular words that networks in the regular and delay conditions found difficult to encode and classify, as well as to understand why networks found these words difficult.

### Item-Specific Effects

**Figure 6 fig6:**
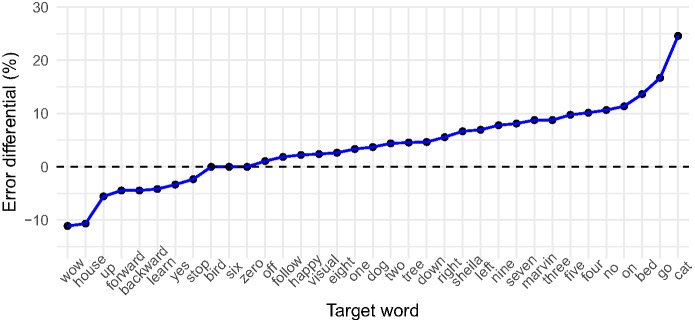
Item Accuracy Differential *Note*. All 35 words from the speech commands data set are shown along the *x*-axis. The error differential is shown on the *y-*axis. A positive differential value signals an advantage (as a percentage accurate) for the networks in the regular maturation condition. A negative differential value signals an advantage for the networks in the delay condition. See the online article for the color version of this figure.

We began our item-specific analyses by computing a by-item accuracy differential, calculated by subtracting the average percentage accurate at test for each word in the delay condition from the average percentage accurate for each word in the regular condition. The result is shown in Figure 6. Here, a positive value indicates a performance advantage, as a percentage, for the regular network, and a negative value indicates a performance advantage for the delay network. Zero differential indicates no performance difference between conditions with respect to a particular word.[Fig fig6]

Networks in the regular condition outperformed networks in the delay condition with respect to 24 out of 35 words, sometimes reaching a differential of 24.6% (for the word cat). Networks in the delay condition, in contrast, performed better on eight words, with a maximum differential of −11.11% for the word wow. Clearly, then, error rates vary as a function of the target word. To better understand these effects, we looked at confusion matrices for predictions made during speech classification in each condition. The top 10 most confused words in the regular and delay conditions are presented in Tables 1 and 2, respectively. These tables show the true word, the total number of misclassifications of that word, the most common misclassification of that word, the number of times that the most common misclassification occurred, and most common misclassification as a proportion of total misclassifications (%).[Table tbl1][Table tbl2]

In many cases, the phonological overlap likely responsible for the misclassification is clear, for instance with respect to tree and three or no and go, and it is noteworthy that networks struggled by some margin with respect to these particular competitor words. Similar patterns are discussed by Karimi and Diaz (2020) who review classification disadvantages for near neighbors under certain experimental conditions. At first glance, then, networks appear to be broadly sensitive to similar spectral features input as human listeners (e.g., struggling with items like tree and three). Yet, Tables 1 and 2 also illustrate examples, which apparently deviate from this pattern, for instance the apparently high rates of misclassification of the word five as the word on or the misclassification of the word house as off. It is difficult to imagine this pattern performance in human participants, and this may attest to the fact that despite the many gross similarities between processing in artificial neural networks and the human brain, artificial neural networks may attend to different features of the input in the service of reducing error in a given task. We return to this point below.

To further understand the above disparities in item accuracy between conditions, we fitted a Bayesian regression model in which test phase accuracy (as a percentage) was predicted by standardized frequency and phonological distance, both in interaction with condition (i.e., regular, delay). We centered on frequency and phonological distance as predictor variables given their importance in the child language literature. However, alternative predictor variables of interest (e.g., orthographic word length) can be experimented with using the Jupyter Notebook and R script associated with this project. Frequency quantified the number of times that a word appeared in the randomly sampled training data. Meanwhile, phonological distance was computed as the mean optimal string alignment distance between a phonological transcription of each target word and of all other words in the speech commands corpus.

**Figure 7 fig7:**
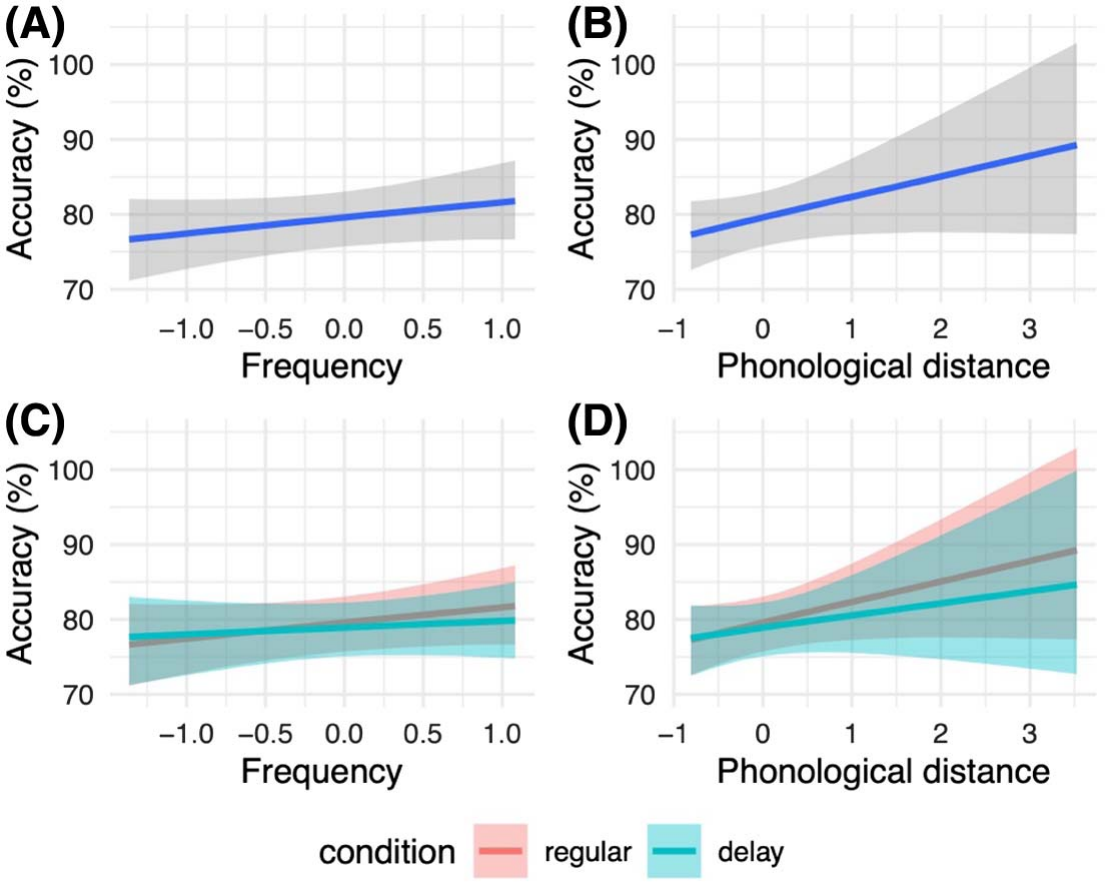
Estimates From a Bayesian Model of the Influence of Frequency and Phonological Similarity on Speech Classification Accuracy *Note*. See the online article for the color version of this figure.

A range of diagnostics showed that this simple regression model with a skew normal likelihood and weakly informative priors fitted well (i.e., rhats at 1.0, a large number of effective samples, and credible posterior predictive checks; see the public repository associated with this project at https://osf.io/x2h8k/ and the brms documentation for further details; Burkner, 2017). Figure 7 shows the estimates from our Bayesian model.[Fig fig7]

In Figure 7, Panels A and B show that across groups, classification accuracy was on average higher for high frequency (β = 2.11; 95% CI [−0.97, 5.45]) and phonologically distinctive (β = 2.82; 95% CI [−0.46, 6.46]) words. While the credible intervals (CIs) associated with these estimates cross zero, indicating that zero may be the true effect, a substantial proportion of probability mass is positively assigned, suggesting that a positive association is likely. Meanwhile, in Figure 7, Panels C and D show that these effects interact slightly with condition but tend in the same positive direction (see R code for full estimates: https://osf.io/x2h8k/). In each case, networks with rapidly maturing high-resolution cochlea models benefitted slightly more from high frequency and greater phonologically distinctiveness.

**Figure 8 fig8:**
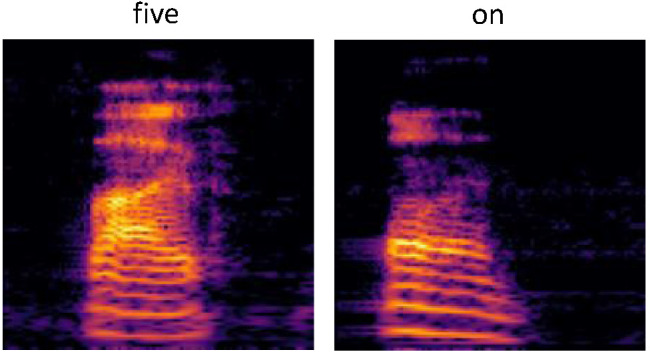
Representative Cochleagrams of the Words Five and On *Note*. See the online article for the color version of this figure.

In summary, item-specific analyses indicate that while networks struggled to different degrees with different words, they nevertheless struggled with broadly similar features of the data set, misclassifying close competitor words such as tree and three most frequently and performing best when words were highly frequent in the training data and phonologically distinctive. Higher resolution low-level auditory representations enabled networks in the regular condition to better exploit these input statistics. These results may be expected given that at any particular period, the regular and delay networks sit at different points on the same developmental trajectory. The resulting performance profiles are in agreement with the general observation that the language of children with DLD is delayed rather than deviant (Kan & Windsor, 2010; see also Discussion section). That is, the language of children with DLD is often similar to that of younger children with typical language skills (though see Bishop, 2013). That said, our item-specific analysis also revealed potential discrepancies between artificial neural network performance and human performance. For instance, we observed a high rate of misclassification of exemplars of five and on (see also the house and off misclassification rate), which at face value would appear unlikely in human participants. If, however, we look at representative raw cochleagrams of the words five and on, for instance, these classification errors perhaps make more sense (all cochleagrams can be visualized using the associated scripts). The distributions of energy in the exemplars shown in Figure 8 are at least visually quite similar and would of course be even more similar were we depreciate their acuity across the *y*-axis (for reference, compare the spectral profiles of five and on to the quite different profile shown for tree in Figure 3).[Fig fig8]

Viewing Figure 8, it may appear reasonable that an artificial neural network would misclassify degraded instances of five and on. But how about a human? Of potential relevance when considering this question is a large research literature looking at so-called adversarial examples. These are stimuli which, when noise that is typically imperceptible to humans is added, result in the radical misclassification of those stimuli in an otherwise high-performing network (Goodfellow et al., 2014; of course, the *y*-axis blur in our study would be perceptible to listening humans). For instance, an image of a panda with visually imperceptible noise added to it may be misclassified as a gibbon by certain artificial neural networks. Understanding adversarial examples is a vital part of research on human and machine learning alignment because it throws light on the marginal disparities between biological and artificial systems that in many other ways appear to perform similarly. Intriguingly, there is limited evidence that the same adversarial examples that derail artificial neural network performance may also affect human performance, just to a lesser extent and emerging in metrics of classification confidence such as response time rather than in raw error rates (Elsayed et al., 2018). Two possibilities, then, are that either the five and on misclassification error and similar striking errors seen in the current simulations are evidence of the inescapable disparity between artificial and biological auditory perceptual processing systems, or, on the other hand, that we might be able to elicit similar patterns of classification behavior (e.g., extended response times) in humans using similar stimuli. There is a precedent for this type of work in the domain of visual processing (Elsayed et al., 2018) but a similar experiment in the domain of auditory processing was outside the scope of the current project.

### Visualizing Internal Representations—Mean Field Theory-Based Manifold Analyses

The cochlea models that provide input to the deep convolutional neural networks used in these simulations were scheduled to mature according to one of two developmental time courses. In contrast, the neural networks into which cochleagrams were passed were provided with a randomized initial weight matrix, which was matched across networks and conditions, but which then optimized freely to solve the specific problems of speech encoding, recognition, and retrieval (note that the control network presents an optimal system, which is free to optimize in the absence of any significant low-level constraint). The performance profiles detailed above—specifically the disparities in accuracy, probability, entropy, and item-specific effects—point to systematic differences in dynamic optimization that, given matching across networks, can result only from these low-level maturational constraints on high-resolution frequency tuning. We are, therefore, modeling discrepancies in optimal adaptation in the face of different low-level constraints. But what does optimization in the face of a low-level frequency discrimination deficit look like? To better understand the optimization profiles of networks in our three conditions and therefore to unpick the representational basis of the performance discrepancies seen in networks across these conditions, we turned to mean field theory-based manifold analyses.

Variables of primary interest were (a) manifold dimensionality and (b) classification capacity. Manifold dimensionality quantifies how spread out through a neural state space long-term speech representations are—that is, how many artificial neurons (as a proportion of the layer size) are implicated in the representation of that speech string. Classification capacity quantifies the number of speech manifolds that can be linearly separated from all competitor representations, again standardized by network layer size. Analysis of biological and artificial neural networks suggests that dimensionality decreases across the auditory and visual perceptual systems, and accordingly, that system capacity increases (Chung & Abbott, 2021; Chung et al., 2018; DiCarlo & Cox, 2007). This transformation reflects the gradual denoising of neural representations in a perceptual hierarchy. Speech representations, for instance, are shown to become decreasingly noise sensitive and increasingly speech selective during transformation from the basilar membrane to the peripheral auditory cortex and beyond (Davis & Johnsrude, 2003; DeWitt & Rauschecker, 2012; Kaas et al., 1999; Okada et al., 2010).

**Figure 9 fig9:**
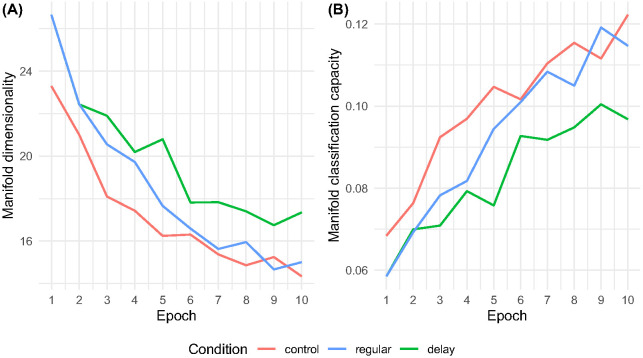
Changes in Manifold Dimensionality and Classification Capacity During Training *Note*. See the online article for the color version of this figure.

System classification capacity has been interpreted as a measure of not only representation overlap, but also of attention or working memory load, given that calculating classification capacity involves linearly discriminating discrete representations from the system’s “long-term memory” in a manner continuous with cognitive recognition and retrieval (Jones & Westermann, 2022). This view is in line with so-called state-based frameworks in which working memory is understood as activated long-term memory that must be optimized to “fit” within an attentional spotlight (Adams et al., 2018; Oberauer, 2013, 2019). Importantly, reducing manifold dimensionality in order to boost system classification capacity is a product of training in a given task, here speech encoding and classification. Training the same artificial neural network with the same data in a different task, for instance a speaker recognition task, would result in an internal network structure optimized for this task (i.e., activation patterns forming manifolds aligned with speaker voice characteristics; Stephenson et al., 2020). The result of this task-specific optimization process is presented in Figure 9, which shows the average manifold dimensionality and classification capacity in networks’ penultimate layers antecedent to the classifier (see Figure 2) as a function of training epoch.[Fig fig9]

Figure 9 shows a clear disparity in the optimization of internal speech representations across conditions. Over 10 epochs, networks following the regular cochlea maturation schedule increasingly approached control standards of optimization supporting low-dimensional representation (Figure 9A). In contrast, despite an overall decrease across epochs, the average dimensionality of internal spoken word representations formed in networks in the delay condition remained significantly higher, that is, these representations were substantially more “spread out” in a relatively poorly optimized neural state space (Figure 9A). In Figure 9, Panel B shows that this inability to optimize efficiently and reduce manifold dimensionality had a severe effect on the delay networks’ ability to retrieve any single representation from their internal “long-term memory” systems—what we interpret here as a form of simulated working memory or attentional capacity deficit. In essence, the delay networks optimized to noise, and this means that the artificial neural response patterns underpinning the long-term representations of different spoken words intersect substantially, making efficient recognition and retrieval difficult. Graphically, it is as though the delay networks remain in the suboptimal state shown in Figure 4C rather than approaching the relatively optimal state shown in Figure 4D alongside networks in the regular and control conditions.

**Figure 10 fig10:**
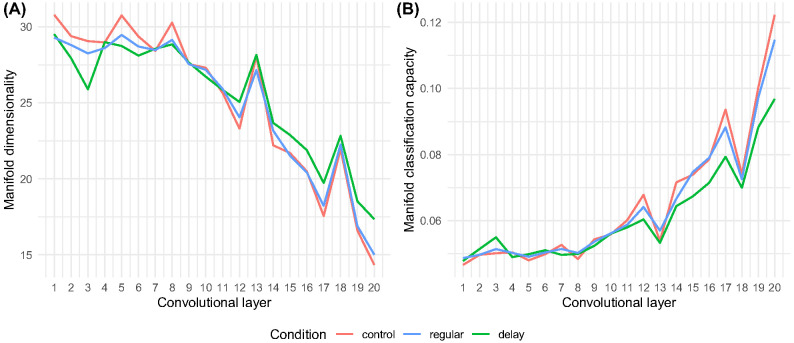
Manifold Dimensionality and Classification Capacity Across the Layers of Trained Networks *Note*. See the online article for the color version of this figure.

The same representational disparity can be seen posttraining across the networks’ layers. In Figure 10, we show the previously reported trajectory (e.g., Yamins & DiCarlo, 2016) across the auditory processing hierarchy from high-dimensional manifolds in a low-capacity system to low-dimensional manifolds in a high-capacity system. Again, this reflects the system optimizing to render initially noise-sensitive representations (i.e., waveform representations containing speaker effects, etc.) increasingly speech selective (i.e., word type representations in the 35-word lexicon). There is, however, a clear optimization disparity between networks in the delay condition and networks in the control and regular conditions in terms of both dimensionality and classification capacity at higher levels of the processing hierarchy. This again demonstrates that due to maturational constraints in the cochlea model, networks in the delay condition failed to learn those spectral features of the speech input that are essential to effective speech encoding, recognition, and retrieval, with noise permeating the system and attentional capacity overwhelmed accordingly (Figure 10B). This can be seen most clearly in Figure 10 with respect to layers 19 and 20, where delay networks deviate sharply from the regular and control networks with respect to both dimensionality and classification capacity.[Fig fig10]

**Figure 11 fig11:**
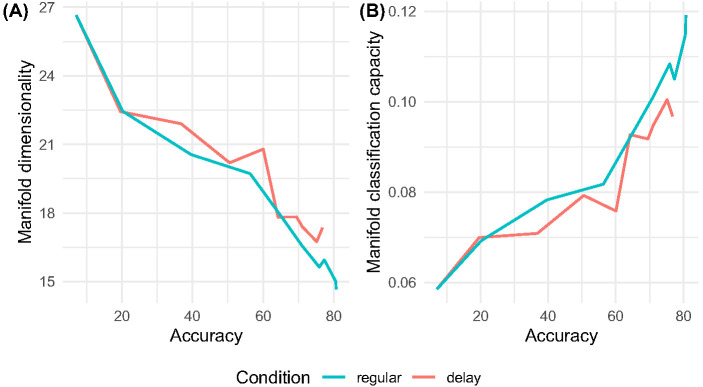
Manifold Dimensionality and Classification Capacity by Performance Accuracy *Note*. See the online article for the color version of this figure.

Finally, we observed optimization disparities even when regular and delay networks were matched on performance accuracy. In Figure 11, we show manifold dimensionality and manifold classification capacity as a function of training phase accuracy by network condition. As in Figure 9, manifold dimensionality and classification capacity are computed for the networks’ final convolutional layer (see Appendix), which is antecedent to the 35-way classifier. Despite very occasional overlap, manifold dimensionality is high and classification capacity is low in the delayed networks relative to the regular networks even when networks in these conditions perform with similar accuracy. This result demonstrates the importance of scrutinizing the internal representations that artificial neural networks form. Based on accuracy alone, we may have wrongly inferred that networks were achieving that level of performance in the same task in the same way, overlooking important differences in the standards of internal optimization. The finding of representational deficits despite matched levels of performance echoes Bishop and McArthur’s reports of electrophysiological discrepancies between children with and without DLD even when DLD group performance is at threshold (Bishop & McArthur, 2005; McArthur & Bishop, 2004; see also Mengler et al., 2005) and Chonchaiya et al.’s (2013) evidence that signatures of poor auditory brainstem optimization are predictive of language outcomes. This reaffirms the important point that apparent successes in task performance may not be underpinned by similar qualities of learning, a point also made by McMurray et al. (2012).[Fig fig11]

In summary, these simulations illustrate how dynamic adaptation to biologically plausible models of cochlea function that mature at different rates results in different optimization profiles, which underpin disparities in key performance metrics (i.e., accuracy, maximum probability assignment, and entropy) and which are evident despite performance accuracy matching (Figure 11). By constraining the development of high-resolution frequency tuning we curtailed the systems’ ability to optimize to encode the key spectral features of the speech input that are integral to solving the task at hand, namely, speech recognition and retrieval. The performance of networks in the delayed condition in this study motivates the prediction that the optimization profile of a biological speech encoding system with a low-level frequency discrimination deficit will show high-dimensional higher-order speech representations (i.e., relatively dispersed neural activation patterns on exposure to speech stimuli), which intersect with competitor speech representations and which are, therefore, not amenable to forming an effective focus of attention. Apparent attention deficits then emerge as a result of attention being thinly spread across imprecise activated long-term speech representations rather than attention being atypically capacity limited. Prior work involving typically developing adults has shown that this prediction regarding divergent neural activation patterns is in principle testable in language disordered populations (Davis & Johnsrude, 2003; DeWitt & Rauschecker, 2012; Kaas et al., 1999; Okada et al., 2010). Indeed, as described in our literature review, there is already some evidence from language disordered populations that is broadly continuous with this claim. For instance, low-quality, “fuzzy,” speech representations are well documented in the behavioral literature looking at children with DLD (Claessen et al., 2009, 2013; Claessen & Leitão, 2012a, 2012b), and atypical neurophysiological signatures indicating suboptimal auditory pathway optimization that is predictive of language impairment have been reported in a number of studies (Bishop & McArthur, 2005; Chonchaiya et al., 2013; McArthur & Bishop, 2004).

## Discussion

Frequency discrimination deficits are widely recognized among children with language learning difficulties (Bishop & McArthur, 2005; McArthur & Bishop, 2004; Mengler et al., 2005). Yet, the nature of these deficits and their relation to speech processing problems remain unclear. The neural microarchitecture supporting high-resolution frequency discrimination matures from the prenatal period through to later childhood, and it is possible that the frequency discrimination deficits seen among some children with language learning difficulties stem from a disruption to this typical developmental trajectory (Bishop & McArthur, 2005; McArthur & Bishop, 2004). Given that frequency tuning throughout the auditory pathway is predominantly attributable to the structural properties of the basilar membrane (i.e., the membrane’s mechanical gradient, including fiber diameter, density, and regularity; Tani et al., 2021), we hypothesized that disruption to the maturation of the structural properties of the basilar membrane may provide a good starting point for inquiry into the source of frequency discrimination deficits in children with neurodevelopmental disorder. Disruption to the structure of the basilar membrane has been demonstrated empirically in animal models manipulating *emilin2* expression, which results in a deficient mechanical gradient and therefore suboptimal functioning of the auditory pathway not supporting high-resolution frequency processing (Amma et al., 2003; Russell et al., 2020).

We developed this theoretical account through a series of computational simulations of speech encoding, recognition, and retrieval. The networks used in these simulations incorporated inner ear models developed to replicate human cochlea function (McDermott & Simoncelli, 2011) that were fed into deep convolutional neural networks. Despite many important differences, for instance in scale, complexity, and the use of undifferentiated cell types, deep convolutional neural networks have demonstrated significant correspondences with human behavioral and neural responses across a range of tests of audition, including speech localization, pitch perception, and hearing in noise (Francl & McDermott, 2022; Kell et al., 2018; Saddler et al., 2021). Our own innovation was to configure the cochlea models that formed a fundamental component of our networks to mature according to different developmental trajectories (i.e., baseline or optimal, regular, and delayed) and to analyze how the subsequent auditory-linguistic pathway optimized in the service of speech encoding, recognition, and retrieval.

Our analysis of networks in the delayed cochlea maturation condition qualitatively replicated the linguistic behavior and neurophysiology of individuals with language learning difficulties in a number of ways, showing (a) delayed acquisition profiles (Norbury et al., 2016), (b) lower spoken word recognition accuracy (Andreu et al., 2012; Evans et al., 2018; Rispens et al., 2015; Velez & Schwartz, 2010), (c) word finding and retrieval difficulties and uncertainty even when performing accurately, as evidenced, for instance, in eye tracking paradigms (i.e., Kambanaros et al., 2015; McMurray et al., 2019; Messer & Dockrell, 2006), (d) “fuzzy” long-term speech representations (Claessen et al., 2009, 2013; Claessen & Leitão, 2012a, 2012b) and neurophysiological signatures of immature neural optimization that are associated with speech and language difficulties (Bishop & McArthur, 2005; Chonchaiya et al., 2013; McArthur & Bishop, 2004), and (e) apparent working memory and attention deficits that are attributable, we believe, to the imprecision of long-term speech representations (Gray et al., 2019; Henry & Botting, 2017; Jones & Westermann, 2022). Our results illustrate that optimizing to low-level, low-resolution spectral representations significantly curtails the capacity of the system to form speech representations supporting efficient recognition and retrieval.

We see, then, that some of the mechanisms widely thought to play a causal role in speech and language disorder may “come for free” if we assume a low-level frequency discrimination deficit. This includes not only the hypothesized working memory capacity bottleneck (Archibald & Gathercole, 2006), which dominates DLD research but which we have argued to be a possible epiphenomenon (see also Jones & Westermann, 2022), but also the so-called lateral inhibition deficit suggested by McMurray et al. (2019). McMurray et al. (2019) argued that a key feature of early language disorder may be an inability to inhibit activated competitor representations during speech recognition and retrieval. Our simulations suggest, however, that an apparent lateral inhibition deficit may be an emergent characteristic of a suboptimal auditory processing hierarchy. Networks in the delayed cochlea maturation condition of our simulations uniformly output predictive distributions with high spread (i.e., high entropy) and low maximum probability assignment, signaling heightened uncertainty and broader activation of the lexicon in response to speech stimuli. As in the case of the hypothesized working memory capacity limitation, then, we believe that evidence offered in support of a deficit in a functionally discrete lateral inhibition mechanism may instead reflect target isolation being overwhelmed due to the imprecision of activated long-term speech representations, a process illustrated in Figure 4C.

**Figure 12 fig12:**
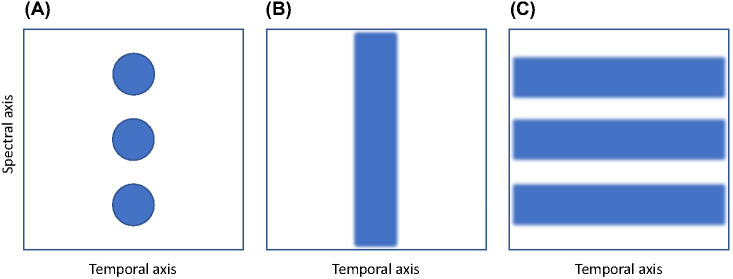
Degrading Critical Features *Note*. See the online article for the color version of this figure.

It may be argued that the results reported in the present study were inevitable. That is, disrupting the quality of the cochlea representations that networks could form would necessarily lead to worse performance. But this is not the case. Indeed, data disruption, for instance blurring, skewing, recoloring, or clipping the training data, is regularly used in machine learning, where the process is termed “data augmentation”, to boost network performance by preventing overfitting and attenuating attention to consistent features (Chollet, 2021). The discrepancies in network performance seen in the present study are, therefore, attributable to the specific features that we degraded—that is, frequency information distributed across the *y*-axis—being essential to the efficient encoding and therefore recognition and retrieval of natural speech. Feature importance is graphically illustrated in Figure 12. In Panel A, we show three dots, exemplifying schematic features that may help us to classify a particular stimulus. In our case, the dots in Figure 12 represent the distinctive frequency components of a speech string. If, as seen in Panel B, we were to degrade this stimulus across the *y*-axis (i.e., the frequency dimension), this would—as demonstrated in the present study—cause problems in determining the identity of that stimulus. On the other hand, degrading the same stimulus across the *x*-axis (i.e., the temporal dimension) preserves the stimulus’ critical features.[Fig fig12]

That is not to say that the *x-*axis degradation seen in Figure 12, Panel C, has no effect. Indeed, work by Saddler et al. (2021) and Saddler and McDermott (2022) has shown that manipulating auditory nerve firing rates to degrade temporal information has a significant negative effect on sound localization and voice recognition. The point is, then, that when it comes to encoding speech efficiently specifically for the purposes of accurate recognition and retrieval, low-level auditory representations with high-resolution, discrete frequency components are particularly important. As we have highlighted throughout this article, there is good evidence that high-resolution frequency discrimination is a core problem among some children with language learning difficulties.

The above discussion of the concept of feature importance may bring some light to the debate regarding whether the auditory processing deficits seen among some children with neurodevelopmental disorders are spectral (i.e., frequency-based) or temporal in nature. As discussed in our Introduction, the early dominant view in DLD research was that the performance deficits seen are temporal in nature, but this view has weakened considerably in the face of failed replications (Bishop & McArthur, 2005; McArthur & Bishop, 2004; Strong et al., 2011; see Rosen, 2003, for review). In contrast, there is compelling evidence that the auditory processing deficits seen among some children with language problems are spectral in nature (Bishop & McArthur, 2005; McArthur & Bishop, 2004; Mengler et al., 2005). Computational simulation indicates that both spectral and temporal information are crucial to effective speech processing, but that the relative importance of these cues is differentially weighted as a function of the task. Temporal acuity is vital, for instance, in the context of voice recognition and sound localization (Saddler et al., 2021; Saddler & McDermott, 2022). Yet, when it comes to encoding speech for the purposes of recognition and retrieval, the current simulations show that high frequency component acuity is key.

It may also be argued that, had we allowed the cochlea models of our delayed networks to continue maturing until they reach the same standard as the cochlea models of our regular networks, network optimization and therefore task performance may have eventually normalized. This is true and reflects the fact that artificial neural networks are not bound by any hard and fast sensitive period or maturational constraints.^3^ Language problems are, in contrast, often evident across the lifespan, suggesting long-lasting disparities in the organization of neural substrates supporting audition and speech. Accordingly, if we take a maturational view of frequency discrimination and speech and language deficits, the critical questions are when and how the typical dynamic adaptation of the auditory pathway becomes “frozen” in a suboptimal state. This appears particularly puzzling given that the auditory pathway is, in general, highly plastic, for instance often adapting quickly to the fitting of a cochlear implant (e.g., Wang et al., 2021). One possibility is that the mechanical gradient of the basilar membrane (and, therefore, tonotopic sensitivity in membrane posterior structures) never reaches optimal differentiation, as in our delayed networks. However, the locus of deficit may of course reside in any of the structures posterior to the cochlea that also support tonotopic mapping. For instance, Bishop and McArthur (2005) noted that while the cochlea is typically fully developed by full-term birth, the auditory brainstem and subsequent structures continue to adapt through childhood, with frequency discrimination skills improving accordingly. Bishop and McArthur (2005) hypothesized, therefore, that either (a) the delayed optimization of higher level structures within the auditory pathway, including the auditory cortex, may be protracted and then plateau with the onset of puberty or (b) that structures of the auditory pathway that support high-resolution frequency tuning may develop slowly but nevertheless fully, yet the cost of a protracted period of maturation during the initial phases of language development may be long lasting.

In this study, we have demonstrated how the auditory pathway may optimize in the face of a cochlea maturation deficit. The basilar membrane remains in our view a good starting point for future inquiry because the deficits we see among some children with DLD are spectral in nature and because the basilar membrane is the seat of tonotopic organization throughout the auditory pathway. We also hypothesized that, given that *emilin2* plays an important role in the emergence of the development of the mechanical gradient of the basilar membrane (Amma et al., 2003; Russell et al., 2020; Tani et al., 2021), potential disruption to the expression of this gene might be considered (though we cite the *emilin2* literature primarily to emphasize how a genetic abnormality can in principle disrupt the emergence of the mechanical gradient of the basilar membrane). Yet, given the enormous complexity of the auditory pathway, numerous possibilities obviously remain. If, through empirical testing, a maturational account is ruled out, it will be necessary to look beyond an early “freezing” of cochlea, auditory brainstem, and auditory cortex maturation, and to instead identify deviances in auditory pathway development that could give rise to low-resolution frequency processing, for instance testing for midfrequency sensorineural hearing loss (i.e., “cookie-bite” hearing loss; see Ahmadmehrabi et al., 2022, for an adult study) that signals potential problems with the cochlea or auditory nerve or identifying cortical dysplasia in neural substrates supporting audition and speech (Bishop, 2014). We should, however, be careful not to attribute a problem to the central nervous system that could be explained by processes in the peripheral auditory system. This is because system complexity grows with the increasing interaction between substrates across the ascending pathway. If disrupted cochlea maturation and function is ruled out in future studies (e.g., by studies involving high-resolution scanning and microscopy of the basilar membranes of individuals with language difficulties; see Liu et al. (2015), for related work), we can then in principle “move up” the auditory pathway systematically toward the auditory cortex and language association areas in our attempt to determine the locus of auditory processing and speech problems in children with language learning difficulties.

An important feature of the present study was to let our networks develop over time, using cochlea models that output representations of increasing spectral acuity according to different maturational trajectories (Figure 3). This developmental approach to modeling with neural networks is uncommon, though it is continuous with a limited number of connectionist studies that have let their networks develop as a function of experience (e.g., Elman, 1993; Westermann et al., 2006; Westermann & Ruh, 2012). We believe that such an approach is integral to the study of the developing brain and mind. Similar work is being conducted by Skelton (2022) who has developed a filter to simulate changes in the visual system during the neonatal period and infancy, which can be used in both experimental stimulus design and in computational models of neurocognitive development. This development-driven approach to computational modeling is likely to provide us with a much richer understanding of the emergence of human cognitive behavior, relative to methods fundamentally aligned with a-developmental adult norms.

Like any method, the use of artificial neural networks to understand human brain function and behavior has its limitations. Neural networks are, of course, a dramatic simplification of the structure of the human brain, involving drastically fewer cells of (in this case) intially identical, undifferentiated types, with activation functions allowing the communication of real numbers. What is more, biological and artificial neural networks learn differently. For instance, biological neural networks appear not to need thousands of labeled exemplars in order to learn spoken words (Lake et al., 2013; though see Lillicrap et al., 2020, for how the brain might approximate the backpropagation algorithm used in our neural networks). These architectural and algorithmic differences may underpin different performance profiles—the high misclassification rates with respect to five and on in our data might be a case in point here. Nevertheless, gross parallels between human performance and brain function and deep neural network activation patterns and performance have been observed repeatedly (Kell et al., 2018; McDermott & Simoncelli, 2011; Saddler et al., 2021; Yamins & DiCarlo, 2016), and a reasonable qualitative mapping with the empirical data in the present study appears to further support this approach.

Modeling of the form presented here of course constitutes a counterpart to, and not replacement of, human assessment. Modeling forces us to be explicit about our assumptions and—as we have demonstrated—may provide computational insight into the nature of representation, recognition, and retrieval within dynamic systems that have optimized to different fundamental constraints. Of course, further analysis involving humans is vital. There have already been important steps in this direction, with Chonchaiya et al. (2013) showing that neural signatures of immature auditory brainstem organization are indicative of poorer language outcomes—a finding highly in agreement with the hypothesis developed in the current article. To date, however, many studies of children with a diagnosis of DLD have included only rudimentary auditory assessments involving, for instance, backward masking, mismatch negativity, or glide discrimination, which can show significant variability before around 8 years of age (Bishop et al., 2005; Bishop & McArthur, 2005; Sutcliffe et al., 2006). One particularly elegant example of the inadequacy of such approaches comes from research demonstrating that children diagnosed with ADHD can complete pure tone discrimination tasks when taking their medication but not when off their medication (Sutcliffe et al., 2006). This highlights the susceptibility of such tasks to nonauditory perceptual influences, including attention. Given the ubiquity of apparent auditory processing problems not only among children diagnosed with DLD but also across other early neurodevelopmental disorders such as developmental dyslexia, there is strong justification for a large-sample study involving rich early auditory assessments (including, for instance, auditory brainstem responses and extended high-frequency audiometry), longitudinal neuroimaging, and the assessment of later language outcomes.

Finally, a comment on our training and testing data. The speech commands data set was chosen for this project because it is free and openly available and because it is unique in comprising such a large number of natural speech exemplars. One limitation of this resource, however, is that it comprises only 35 word types, meaning that only limited insight can be drawn from our item-specific analyses. While we believe that the use of the speech commands data set in the current project is well justified, going forward it would be useful to replicate our findings using a larger data set. In particular, it would be valuable to test children and artificial neural networks using the same speech stimuli, which could be recorded specifically for this purpose. This would support a relatively direct comparison between child and artificial neural network behavior. Indeed, using this approach, it would be possible to simulate real-world language interventions and to determine the computational basis of their efficacy.

## Conclusion

Frequency discrimination is a core problem for many children with language learning difficulties and through computational simulation we have shown how this deficit would propagate problems with the encoding, recognition, and retrieval of natural speech. Our simulations provide proof of concept that the optimization of the auditory pathway to low-resolution cochlea representations—part of a typical maturational trajectory that may be disrupted in DLD—results in patterns of linguistic behavior that align qualitatively with a range of empirical findings observed among children with DLD. Our speculation that the locus of such deficits may be a disruption to the maturation of the basilar membrane during a sensitive period of auditory pathway optimization reflects the fact that the mechanical gradient of the basilar membrane provides the basis for the emergence of frequency sensitivity across the auditory pathway. Yet, this hypothesis of course requires empirical testing. The auditory pathway is a highly complex system, which could be disrupted at any level. Also in need of further scrutiny is our speculation, given the contemporary animal model literature, that atypicalities in *emilin2* expression may be implicated in the disruption of the emergence of the mechanical gradient of the basilar membrane (i.e., the development of fibril microarchitecture supporting high-resolution processing, which promulgates the required tonotopic sensitivity through the auditory nerve, brainstem, and cortex). We fully recognize these elements of our argument to be speculation, albeit empirically driven speculation. Our view is simply that the empirical evidence with respect to structural changes in the basilar membrane suggests that this hypothesis constitutes a strong starting point for further inquiry into the nature of auditory processing deficits in children with language learning difficulties.

## Figures and Tables

**Table 1 tbl1:** Top 10 Speech Classification Errors in the Regular Condition

Word	Total misclassifications	Most common misclassification	Number	Proportion of total misclassifications (%)
Tree	66	Three	17	25.76
No	141	Go	26	18.44
Follow	54	Four	7	12.96
Go	78	No	10	12.82
Up	72	Off	9	12.5
House	75	Off	8	10.67
Four	69	Forward	7	10.14
Five	123	On	10	8.13
One	90	Nine	7	7.78
Off	93	On	7	7.53

**Table 2 tbl2:** Top 10 Speech Classification Errors in the Delay Condition

Word	Total misclassifications	Most common misclassification	Number	Proportion of total misclassifications (%)
Tree	66	Three	17	25.76
No	141	Go	30	21.28
Go	78	No	13	16.67
Four	69	Forward	10	14.49
Five	123	On	16	13.01
On	132	Five	16	12.12
Right	108	Five	10	9.26
Two	114	Go	10	8.77
Three	114	Eight	9	7.89
No	141	Down	9	6.38

## Footnotes

1 We note that the term “temporal processing deficit” has been objected on the basis that this body of research shows no evidence that the awareness of temporal order is compromised among children with DLD. The assumed difficulty instead relates to rapid changes in frequency and so the term “rapid perception deficit” may be more appropriate (Bishop, 2014, p. 90).

2 This article is associated with a fully annotated Jupyter Notebook (Kluyver et al., 2016), which is available from the following public repository and which can be used to replicate the simulations described or to experiment with alternative parameter configurations at https://osf.io/x2h8k/.

3 That said, we note that sensitive periods may stem from entrenchment rather than biological ossification and can therefore emerge in computational systems (Thomas & Johnson, 2006).

## ResNet-18 Convolutional Layer Specification and Hyperparameters

**Table:**

Layer index	Layer name	Output size	Kernel size	Stride	Padding
1.	Convolutional 2D	1, 64	7, 7	2, 2	3, 3
2.	Convolutional 2D	64, 64	3, 3	1, 1	1, 1
3.	Convolutional 2D	64, 64	3, 3	1, 1	1, 1
4.	Convolutional 2D	64, 64	3, 3	1, 1	1, 1
5.	Convolutional 2D	64, 64	3, 3	1, 1	1, 1
6.	Convolutional 2D	64, 128	3, 3	2, 2	1, 1
7.	Convolutional 2D	128, 128	3, 3	1, 1	1, 1
8.	Convolutional 2D	64, 128	1, 1	2, 2	n/a
9.	Convolutional 2D	128, 128	3, 3	1, 1	1, 1
10.	Convolutional 2D	128, 128	3, 3	1, 1	1, 1
11.	Convolutional 2D	128, 256	3, 3	2, 2	1, 1
12.	Convolutional 2D	256, 256	3, 3	1, 1	1, 1
13.	Convolutional 2D	128, 256	1, 1	2, 2	n/a
14.	Convolutional 2D	256, 256	3, 3	1, 1	1, 1
15.	Convolutional 2D	256, 256	3, 3	1, 1	1, 1
16.	Convolutional 2D	256, 512	3, 3	2, 2	1, 1
17.	Convolutional 2D	512, 512	3, 3	1, 1	1, 1
18.	Convolutional 2D	256, 512	1, 1	2, 2	n/a
19.	Convolutional 2D	512, 512	3, 3	1, 1	1, 1
20.	Convolutional 2D	512, 512	3, 3	1, 1	1, 1
*Note*. See Jupyter Notebook for full network specification.					

**Table:**

Hyperparameters
Optimizer: stochastic gradient descent
Learning rate: .001
Momentum: .9
Loss function: cross-entropy loss
